# *CCR7* Has Potential to Be a Prognosis Marker for Cervical Squamous Cell Carcinoma and an Index for Tumor Microenvironment Change

**DOI:** 10.3389/fmolb.2021.583028

**Published:** 2021-04-01

**Authors:** Wei-Jie Tian, Peng-Hui Feng, Jun Wang, Ting Yan, Qing-Feng Qin, Dong-Lin Li, Wen-Tong Liang

**Affiliations:** ^1^Department of Gynecology, Guizhou Provincial People’s Hospital, Medical College of Guizhou University, Guiyang, China; ^2^Department of Obstetrics and Gynecology, Peking Union Medical College Hospital, Peking Union Medical College, Chinese Academy of Medical Sciences, Beijing, China

**Keywords:** *CCR7*, tumor microenvironment, CIBERSORT, ESTIMATE, tumor-infiltrating immune cells, cervical squamous cell carcinoma

## Abstract

The tumor microenvironment (TME) has an essential role in the development of cervical squamous cell carcinoma (CSCC); however, the dynamic role of the stromal and immune cells is still unclear in TME. We downloaded data from The Cancer Genome Atlas (TCGA) database and applied ESTIMATE and CIBERSORT algorithms to measure the quantity of stromal and immune cells and the composition of tumor-infiltrating immune cell (TIC) in 253 CSCC cases. The protein-protein interaction (PPI) network and Cox regression analysis presented the differentially expressed genes (DEGs). Then, C-C chemokine receptor type 7 (*CCR7*) was screened out as a prognostic marker by the univariate Cox and intersection analysis of PPI. Further analysis showed a positive correlation between the expression of *CCR7* and the survival of CSCC patients. The result of the Gene Set Enrichment Analysis (GSEA) of genes in the high *CCR7* expression group displayed a predominant enrichment in immune-related pathways. An enrichment in metabolic activities was observed in the low *CCR7* expression group. CIBERSORT analysis showed a positive correlation between Plasma cells, CD8^+^ T cells, and regulatory T cells and the *CCR7* expression, suggesting that *CCR7* might play a crucial role in maintaining the immunological dominance status for TME. Therefore, the expression level of *CCR7* might help predict the survival of CSCC cases and be an index that the status of TME transitioned from immunological dominance to metabolic activation, which presented a new insight into the treatment of CSCC.

## Introduction

Cervical squamous cell carcinoma (CSCC) remains to be the second leading cause of cancer death in women, resulting in over 300,000 deaths worldwide (Siegel et al., 2020). Currently, the primary treatment of early-stage cervical cancer is surgery. Nevertheless, for patients with advanced stages, primary therapy is radiotherapy and chemotherapy, and these therapies have met with limited success (Gupta et al., 2018; Cohen et al., 2019; Wright et al., 2019; Grigsby et al., 2020). The main survival indicator for CSCC is the International Federation of Gynecology and Obstetrics (FIGO) stage. However, with the biological heterogeneity of CSCC, Patients may have distinctly various treatment outcomes, albeit they are of the same FIGO stage. Thus, it is essential to identify a new marker reflecting the heterogeneity of CSCC and helping to schedule individualized treatment for patients with otherwise poor survival.

Increasing evidence has shown that the tumor microenvironment (TME) has a vital role in tumor development. Cancer cells collaboratively interacted with their supporting cells, and the interactions determined the malignant characteristics of tumor, such as immortalization, immune escape, and resisting apoptosis. Therefore, TME can influence the therapeutic effect and clinical outcomes of patients with cancer (Bader et al., 2020; Hoffmann and Ponik, 2020). Resident stromal cells and recruited immune cells are mainly structural cell elements of the TME. Meanwhile, studies reported that stromal cells could contribute to extracellular matrix remodeling and tumor metastasis, but the mechanism is still not clear (Vila-Ibarra et al., 2019; Qian et al., 2020).

Of note, many researchers have focused on the role of the immune cells in TME on tumor development. Studies reported that the tumor-infiltrating immune cell (TIC) in TME could serve as a useful marker of the treatment response (Huang et al., 2018; Peltanova et al., 2019). Moreover, an elevated tumor-infiltrating CD8^+^ lymphocyte showed the potential to be an excellent clinical prognostic marker for cervical cancer patients after neoadjuvant chemotherapy (Liang et al., 2018).

Cervical cancer cases with elevated regulatory T cell rate have poorer overall survival (OS) than those with low Treg frequency (Shang et al., 2015). This correlation resulted in the application of immune-based therapeutics and gave rise to the improved immune checkpoint inhibitors for cervical cancer patients (Chung et al., 2019; Kagabu et al., 2019). Studies reported that the immunologic escape of cancer cell in TME occurs before the cervical cancer invasion (Cicchini et al., 2016; Luo et al., 2020). It implied that the immunologic response in TME is exceedingly crucial at the initial development of cervical cancer. However, at present, it is challenging to conduct an accurate search that could precisely reveal the dynamic change of the stromal and immune elements in TME.

Next-generation sequencing technology with genome functional analysis develops rapidly and makes it possible to decipher the functions of various kinds of cells during TME change. Our research used CIBERSORT and ESTIMATE algorithms methods to measure the ratio of stromal cells and immune cells and the TIC proportion of CSCC samples from the TCGA database. We identified a prognostic biomarker, C-C chemokine receptor type 7 (*CCR7*). We explored the DEGs founded by comparison between stromal elements and immune elements in CSCC samples. We also identified that the *CCR7* has the potential to be a useful index to indicate the transformation of TME status in CSCC.

## Results

### The Workflow of This Study

**FIGURE 1:**
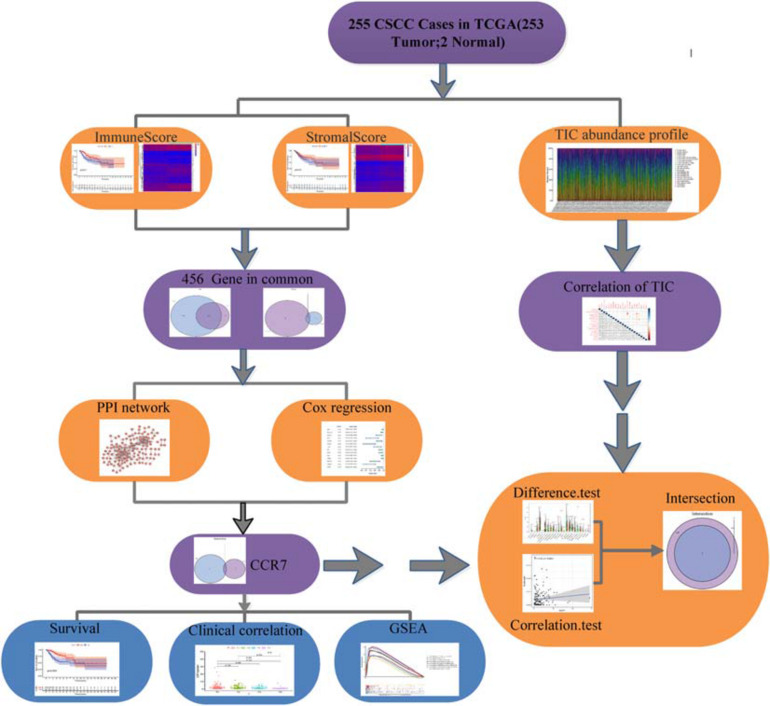
The flowchart of this study.

Figure 1 showed the analysis process of our study. To evaluate the components of TICs and the number of stromal and immune cells in CSCC samples, we downloaded RNA-seq documents of 255 cases from the TCGA database (Supplementary Table 1). DEGs shared by StromalScore and ImmuneScore were presented by univariate Cox regression analysis and applied to formulate a PPI network. Then gene *CCR7* was screened out by intersection analysis using the hub genes in the PPI network and the most critical genes acquired from the study of univariate Cox regression. Then we concentrate on *CCR7* for the following analysis, including survival analysis, immunohistochemistry, correlation analysis with clinicopathological features, correlation with TICs, GSEA, and Cox regression.

### Scores and Prognosis of CSCC Patients

**FIGURE 2:**
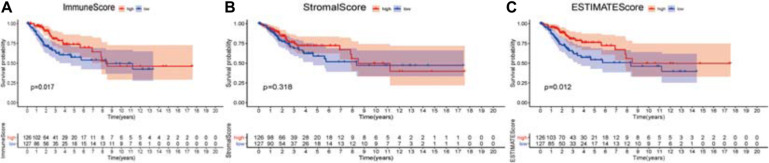
Association of three types of scores with overall survival of CSCC patients. **(A)** High ImmuneScore group correlated with a better prognosis, *p* = 0.017. **(B)** Stromalscore was not associated with overall survival, *p* = 0.318. **(C)** Elevated ESTIMATEScore was associated with better survival, *p* = 0.012. The numbers below the plots indicate the number of patients at risk in each group at the given time points.

The higher scores estimated in StromalScore or ImmuneScore mean the more significant number of the stromal or immune elements in TME. The ESTIMATEScore was the sum of StromalScore and ImmuneScore, indicating the overall ratio of both parts in TME. The portion of immune elements positively correlates with the OS of CSCC patients (Figure 2A). Although there was no correlation between StromalScore and the OS (Figure 2B), ESTIMATEScore still positively correlates with the OS of CSCC patients (Figure 2C). The analysis demonstrated that immune elements in TME were appropriate for indicating the survival of CSCC patients.

### Scores and Pathological Features of CSCC Patients

The correlation between the ratio of stromal and immune components and the pathological characteristics was explored. The results showed that ImmuneScore, StromalScore, and ESTIMATEScore had no correlation with tumor grade and T classification, N classification of TMN stages (Supplementary Figure 1).

### DEGs Shared by StromalScore and ImmuneScore Presented the Enrichment of Immune-Related Genes

**FIGURE 3:**
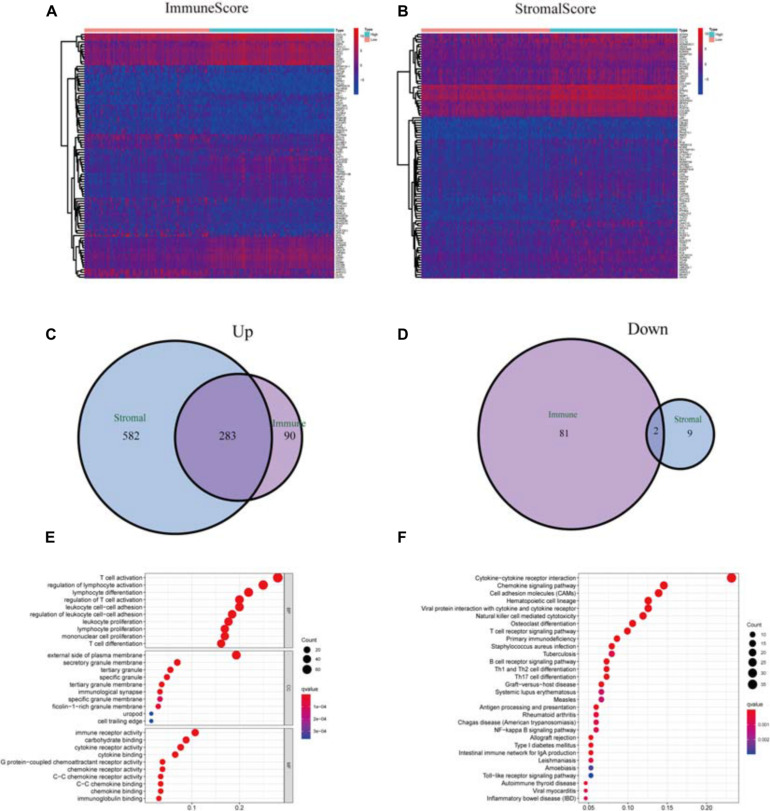
Analysis of DEGs. **(A)** Heatmap of DEGs in ImmuneScore (high score, right; low score, left. | log FC| > 1.5, *q* = 0.01). **(B)** Heatmap of DEGs in StromalScore, similar to heatmap A. **(C)** Venn plots of commonly upregulated DEGs shared by StromalScore and ImmuneScore. **(D)** Venn plots of commonly downregulated DEGs. **(E,F)** GO term and KEGG pathway analysis of the 285 DEGs.

We performed a comparative analysis between low- and high-score samples to determine the exact diversification of gene spectrum in TME regarding stromal and immune components. From ImmuneScore, when compared to the median (high score vs. low score), a total of 456 DEGs were identified: 373 were upregulated, and 83 were down-regulated (Figures 3A,C,D). Similarly, from StromalScore, a total of 876 DEGs were obtained: 865 were upregulated, and 11 were down-regulated (Figures 3B–D). The Venn plot demonstrated the intersection analysis. In StromalScore and ImmuneScore, there were 283 upregulated genes shared by high score samples and two down-regulated genes shared by the low score samples. These 285 DEGs might be significant markers for the condition of TME. Enrichment analysis of gene ontology (GO) demonstrated that most DEGs matched the immune-related GO terms, such as T-cell activation and lymphocyte differentiation (Figure 3E). The analysis of the Kyoto Encyclopedia of Genes and Genomes (KEGG) also showed that most of the DEGs matched the chemokine signaling pathway, hematopoietic cell lineage, and cytokine–cytokine receptor interaction (Figure 3F). Thus, these DEGs seemed to primarily participate in the immune-related pathways, which indicated that immune were vital characteristics of TME in CSCC.

### Univariate Cox Regression Analysis and PPI Network

**FIGURE 4:**
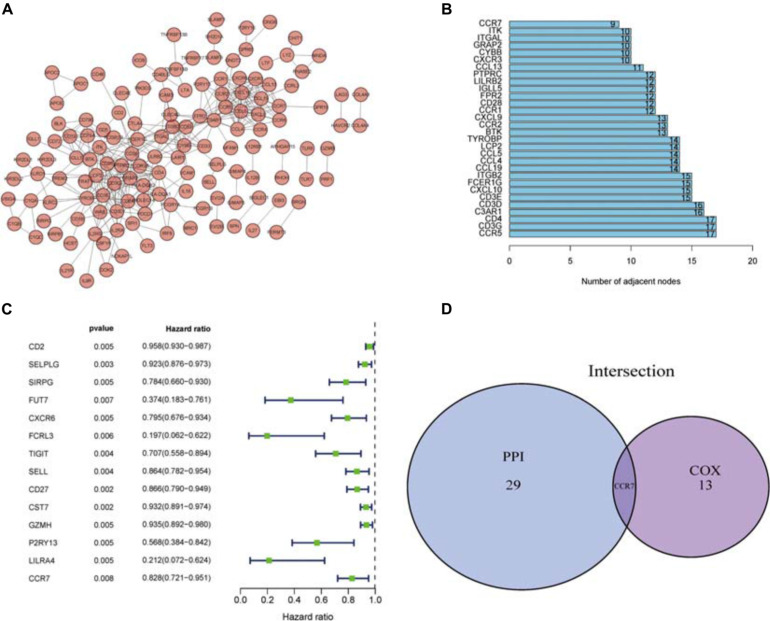
PPI network and univariate Cox analysis. **(A)** The PPI network was constructed using Cytoscape. **(B)** The top 30 genes ranked according to the number of nodes. **(C)** The top significant DEGs with *p* < 0.01 in univariate Cox regression analysis. **(D)** Genes shared by the top 30 nodes and the most significant DEGs in univariate Cox.

To further elucidate the potential mechanism, the PPI network was constructed by using Cytoscape software. Figure 4A showed the interactions between 285 genes, and the bar plots displayed the significant 30 genes selected according to the number of nodes and edges (Figure 4B). The significant factors among 285 DEGs were determined by using univariate Cox regression analysis (Figure 4C). After that, we performed an intersection analysis between the top 14 factors arranged by the significant *p*-value of univariate Cox regression and the topmost nodes in the PPI network. Only *CCR7* overlapped from both analyses (Figure 4D).

### The Relationship Between *CCR7* Expression and the Survival and TNM Stages in CSCC Patients

**FIGURE 5:**
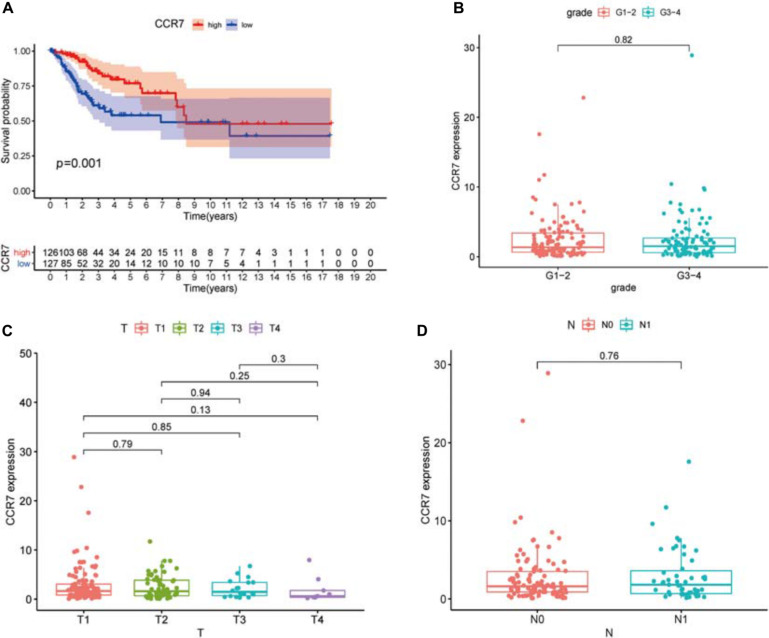
Association between the expression of *CCR7* and survival and clinicopathological features of CSCC patients. (**A**) High *CCR7* expression group correlated with a better prognosis, *p* = 0.001. **(B–D)** The relationship between *CCR7* expression and clinicopathological characteristics.

We compared all CSCC samples with the median of the *CCR7* expression level and divided them into *CCR7* high- and low-expression group. The survival analysis showed that the *CCR7* high expression group had a better prognosis than the *CCR7* low expression group (Figure 5A).

**FIGURE 6:**
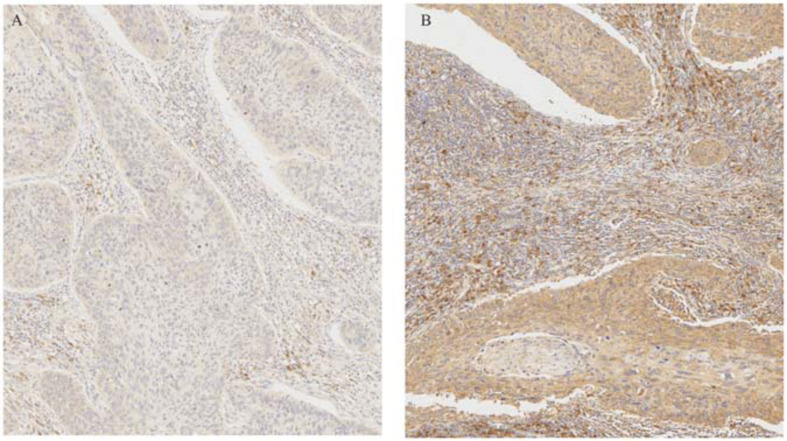
Immunohistochemical staining of CCR7 in cervical cancer specimens and normal tissue. **(A)** Negative CCR7 staining in a squamous cell carcinoma. **(B)** Positive CCR7 staining in a squamous cell carcinoma.

The results demonstrated that the *CCR7* expression level in TME was positively correlated with the survival of CSCC patients. And then, the relationship between *CCR7* expression and clinical characteristics was analyzed. Although the expressions of *CCR7* have no statistical significance between different grade or TNM stages (Figures 5B–D), there was a tendency that the *CCR7* expression level was decreased along with the advancement of the T classification of TMN stages. With only two samples of the normal group, we did not explore the difference in the expression of *CCR7* between tumor samples and normal samples. To further verify the relationship between *CCR7* expression and clinical characteristics, immunohistochemistry and staining evaluation of 70 CSCC tissues and nine normal cervical tissues were done. The CSCC tissues showed a higher *CCR7* expression than normal tissues (Table 1 and Figure 6). There was no statistical significance between *CCR7* expression and stages or grade. However, the tendency that the *CCR7* expression level negatively correlates with the advancement of stages or grade still existed (data not shown).

**TABLE 1:** Association between *CCR7* expression and clinicopathological factors.

Variables	*CCR7* expression		*P* value^*a*^
	
	−	+	
**Age (years)**			
≤45	21	18	>0.05
>45	22	18	
**Histological type**			
SCC	35	35	0.0349
Normal	8	1	
**FIGO stage**			>0.05
I	26	27	
II	3	3	
**Grade**			>0.05
I	2	2	
II	16	18	
III	11	5	

*SCC, squamous cell carcinoma;*

*FIGO, International Federation of Gynecology and Obstetrics.*

*^a^Chi-square test.*

### The Potential of CCR7 to Be a Marker of TME Modulation

**FIGURE 7:**
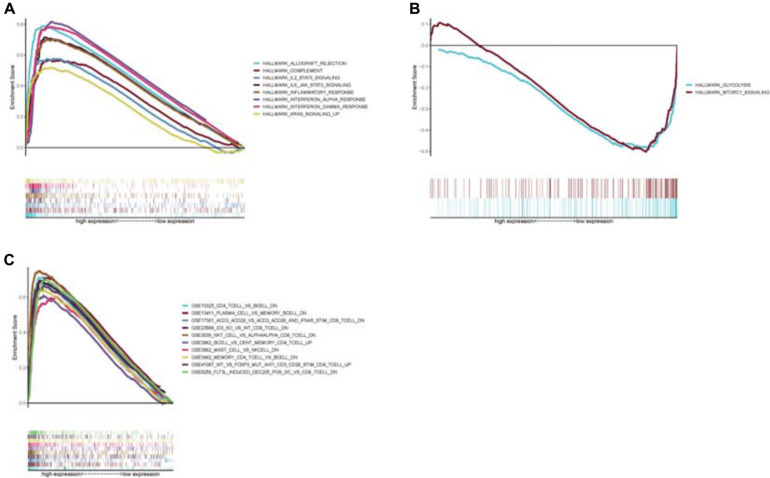
Enrichment plots from GSEA analysis **(A)** GSEA analysis showed that several gene sets were differentially enriched in *CCR7* high expression samples. **(B)** The gene sets enriched in *CCR7* low expression samples. **(C)** The enriched gene sets in C7.all.v7.1.symbols in high *CCR7* expression samples. Only several top gene sets were showed in the chart.

Because the *CCR7* expression level positively correlated with the survival of CSCC patients, we conducted GSEA in the *CCR7* high-expression and low-expression groups, respectively. The genes of the high-expression group of *CCR7* were predominantly centralized in immune-related pathways, such as inflammatory response, allograft rejection, and complement (Figure 7A). In the low-expression group of *CCR7*, several genes were centralized in metabolic activities, including mTORC1 (mammalian target of rapamycin complex 1) and glycolysis (Figure 7B). For the immunologic gene sets of C7 collection defined by MSigDB, several immune-related gene sets were matched to the high expression group of *CCR7* (Figure 7C). However, no gene sets were matched to the *CCR7* low expression group. These results suggested that *CCR7* might be useful to be a marker of the status of TME.

### Relationship Between CCR7 Expression and the Proportion of TICs

**FIGURE 8:**
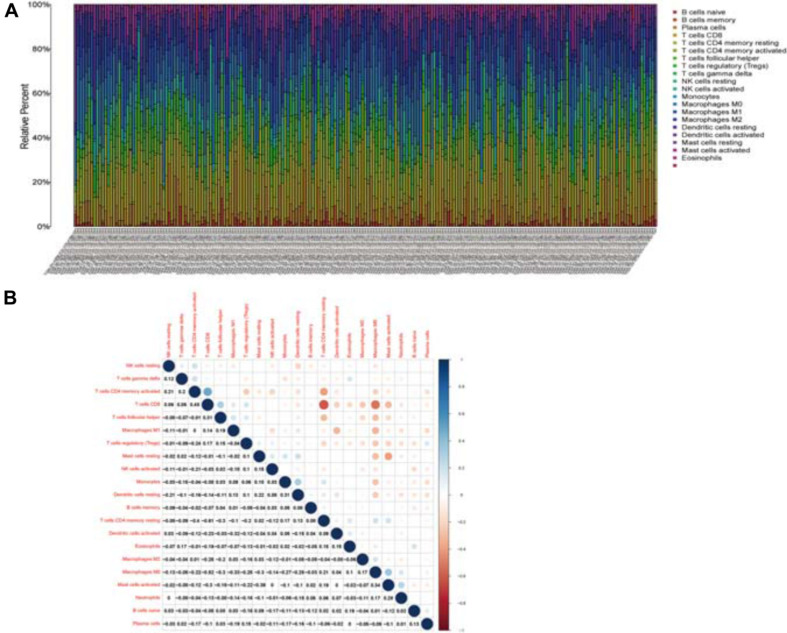
TIC profile and correlation analysis. **(A)** The relative proportions of 21 TICs subpopulation in CSCC tumor samples. **(B)** The correlation matrix of all 21 kinds of TICs.

**FIGURE 9:**
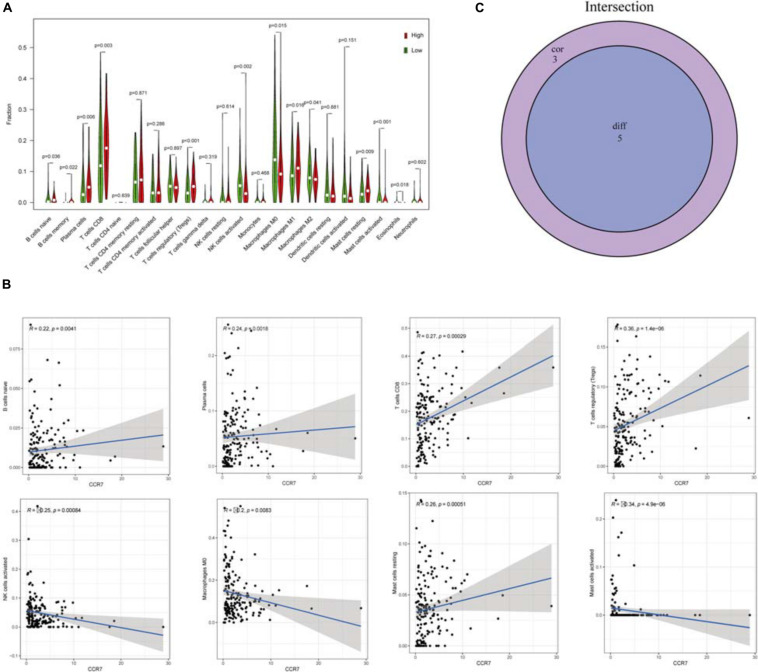
The relationship between TICs proportion and *CCR7* expression. **(A)** The Violin plot exhibits the difference in immune cells between CSCC tumor samples with high or low CCR7 expression. **(B)** The association between 8 kinds of TICs proportion and the *CCR7* expression (*p* < 0.01). **(C)** Venn plot showed that five types of TICs were associated with the expression of *CCR7* codetermined by correlation and difference tests.

Twenty-one subsets of the immune cell were obtained by using the CIBERSORT algorithm to evaluate the components of tumor-infiltrating immune subsets (Figure 8). The intersection of correlation and difference analysis demonstrated that five types of TICs associated with the expression level of *CCR7* (Figure 9 and Supplementary Table 2).

Among them, three types of TICs had a positive correlation with the expression level of *CCR7*, including Plasma cells, regulatory T cells and CD8^+^ T cells; two types of TICs had a negative relationship with the expression level of *CCR7*, including activated mast cells and activated NK cells. The above results demonstrated that *CCR7* has an impact on the immune activity of TME.

## Discussion

In this study, we downloaded data from the TCGA database and explored TME-related genes correlated to the prognosis of CSCC patients. *CCR7* was screened out to be associated with the prognosis of CSCC patients and related to immune activities. Remarkably, the results of bioinformatics analysis demonstrated that *CCR7* could be a marker of the condition of TME in CSCC patients.

It is known that TME played an essential part in the development of tumors (Meurette and Mehlen, 2018). Therefore, it is beneficial to research new therapeutic targets leading to change of TME and contributing to the transformation of TME from tumor-suppressed to tumor-friendly. Many studies had presented the significance of the immune microenvironment in cancer development. This study demonstrated that immune elements in TME related to the survival of CSCC patients. The result showed the vital significance of exploring the mutual effect between immune cells and cancer cells, which brought new insight into establishing a novel, useful treatment paradigm. Significant progress has been obtained in the field of immunotherapy, and the FDA has approved immune checkpoint inhibitors (ICIs) for patients with cervical cancer (Chung et al., 2019).

However, programmed death 1 ligand, the most crucial marker, is unsatisfactory to be an excellent indicator to help clinicians to determine which cervical cancer patients could benefit from the immunotherapy (Wang and Li, 2019). Cervical cancer was a biologically heterogeneous carcinoma and was considered to be non-immunogenic cancer. Nevertheless, some researchers have proved that the consistent existence of human papillomavirus, the primary etiology of cervical cancer, can lead to host immune detection’s shutdown and enable tumor cell’s immune evasion (Luo et al., 2020). Besides, the abundance of TILs was reported to correlate with the prognosis of cervical cancer significantly. The lymphocytes in peripheral blood were significantly lower in cervical cancer patients than that in the precancerous lesion patients (Saglam et al., 2019; Wu et al., 2020). Despite the ICIs demonstrating promising efficacy in cervical cancer treatment, for women with cervical cancer, the clinical effect of immune checkpoint blockade has been modest with single agents, and immune-related adverse reactions also raise concerns (Saglam et al., 2019). Therefore, it is essential to explore new prognostic markers for the immunotherapy of cervical cancer. Here, the study demonstrated that the low expression level of *CCR7* has obviously a correlation with poor prognosis. The results suggested that *CCR7* might be a useful therapeutic target and a prognostic marker for TME in CSCC.

C-C chemokine receptor type 7 is a G-protein-coupled receptor commonly expressed by B cells, mature DCs, CD56^*bright*^ NK cells, and T-cell subset (Sánchez-Sánchez et al., 2004). it is increasingly evident that these receptors control the function of several tumor-promoting processes, including cancer cell growth, metastasis through the recruitment of immune cell subsets, and host immune responses against malignant cells (Nagarsheth et al., 2017). For example, many studies reported that *CCR7* promotes the progression of several different tumors (Heresi et al., 2005; Wang et al., 2005).

CD56^*bright*^/CCR7^+^ NK cells can migrate to the lymph nodes to promote the production of cytokines, but CD56^*bright*^/CCR7^+^ NK cells are less cytotoxic than the CD56^*dim*^/CCR7^–^ subset (Yao and Matosevic, 2021). Central memory and naïve T cells generally express high levels of CCR7. Hence, they can be activated in the lymph nodes and spleen and developed into effector T cells. Effector T cells can move to infected or tumorigenic locus by the downregulation of CCR7 expression (Choi et al., 2020).

However, it is still unclear about the role of *CCR7* during tumor development. Studies reported that the expression level of *CCR7* may be correlated with lymph node metastasis and can serve as a marker of poor prognosis in patients with cervical cancer (Kodama et al., 2007; Dai et al., 2017). In contrast, elevated *CCR7* expression can be an independent prognostic marker for better overall survival in hematological malignancies and breast cancer patients (Li et al., 2017; Ma et al., 2019). Therefore, the function of *CCR7* in tumor still needs to be clarified; it can function pro- or antitumor effects depending on where it is expressed. When expressed in cancer cells, it promotes tumor progression, whereas, in immune cells such as Treg cells and NK cells, antitumor functions are initiated (Pesce et al., 2016; Mollica Poeta et al., 2019).

Besides, it has been reported that *CCR7* might be involved in mediating lymphocyte cell trafficking in TME (Dios-Esponera et al., 2019). Therefore, we investigated the relationship between the expression level of *CCR7* and TME. The GSEA displayed that several immune-related pathways, such as inflammatory response, allograft rejection, and complement, were matched to the *CCR7* high-expression group. While in the *CCR7* low-expression group, metabolism-related activities, such as mTORC1 signaling and glycolysis, were matched. It suggested that *CCR7* might play a part in the state change of TME from immunological dominance to metabolic dominance. This conversion of TME and the reduction of antitumor TICs demonstrated that *CCR7* might restrain the development of tumor in CSCC.

C-C chemokine receptor type 7 was known to be essential for T lymphocyte functions. In this study, the CIBERSORT analysis showed that T cells positively correlated with the expression level of *CCR7* in CSCC patients. In the low-expression group, the mTORC1 signaling pathway was enriched. The classical pathway of mTORC1 participates in regulating the metabolism of T cells. The mTORC1 pathway is activated upstream by phosphoinositol-3-kinase (PI3K) to control the metabolism of cells that regulate the cell fate of T cell subsets (Laplante and Sabatini, 2012). Therefore, the positive relationship between the expression level of *CCR7* and the number of T cells in CSCC patients demonstrated that CCR7 might play an essential role in preserving immune-active status in TME and might potentially be a marker for cancer microenvironment transformation in cervical cancer.

The results also showed that CCR7 expression negatively related to activated NK cells, including “activated” CD56^*dim*^ and CD56^*bright*^ NK cells (Cooper et al., 2001; Pesce et al., 2016). This result was consistent with previous studies that CD56^*dim*^ NK cells are negative for CCR7 and act as the “killers” to directly lysis tumor cells. Of note, in analogy to resting CD56^*dim*^ and CD56^*bright*^ NK cells, resting NK cells expressing CCR7 do not correlate with CCR7 expression in our study. The CD56^*bright*^ NK cells only comprise 10% of all peripheral blood NK cells and populate secondary lymphoid compartments (Cooper et al., 2001). Meanwhile, only 102 cases with resting NK cells abundance profile were included in the correlation analysis. The low number of resting NK cells and samples may lead to a negative result.

Using bioinformatics analysis, we explored the TME-related genes in CSCC. *CCR7* might be a useful prognostic marker for CSCC patients. Moreover, *CCR7* could be a useful marker for the change of TME condition from immunological dominance to metabolic dominance. However, further researches are necessary to elucidate the accuracy of a combined analysis of the expression level of *CCR7* and the number of tumor-infiltrating T-cell in CSCC patients. Meanwhile, it is significant to investigate the roles of different subpopulations among T or NK cells which may correlate with the expression level of *CCR7*.

## Materials and Methods

### Raw Data

We downloaded the RNA-seq data and the corresponding clinical data of 255 CSCC cases (253 tumor samples; 2 normal samples) from the TCGA database with level 3.

### Calculation of StromalScore, ImmuneScore, and ESTIMATEScore

We downloaded the R script of ESTIMATE from the public source website^1^ to evaluate the proportion of stromal-immune ingredient in TME for every sample, presented in three types of scores: StromalScore, ImmuneScore, and ESTIMATEScore (Yoshihara et al., 2013), which represented the proportion of stromal cell, immune cell, and the sum of both cells, respectively. It indicates the more considerable the amount of the component in TME, the higher the corresponding score.

### Survival Analysis

We performed a survival analysis by R software with R package survival and survminer. With a survival period from 0 to 17.6 years, two hundred fifty-three tumor samples had detailed survival data. The survival curve was produced by Kaplan–Meier method and analyzed by the log-rank test. Two-tailed *p* < 0.05 was considered statistically significant.

### DEGs Between High- and Low-Score Groups of StromalScore and ImmuneScore

According to the median of StromalScore and ImmuneScore, we grouped the 253 tumor samples into a high- or low-score group, respectively. DEGs were screened out by comparing the high-score samples with the low-score samples through differentiation analysis of the gene expression (Smyth et al., 2005). DEGs were considered significant if false discovery rate (FDR) < 0.01 and the fold change > 1.5.

### Heatmaps, GO, and KEGG Enrichment Analysis

By using R language software with package pheatmap, heatmaps of DEGs were displayed. By packages enrichplot, ggplot2, and clusterProfiler, GO, and KEGG enrichment analysis were conducted. Both *p*- and *q*-value of less than 0.05 were considered significantly enriched (Ashburner et al., 2000; Kanehisa et al., 2017).

### Correlation Between Scores and Clinical Stages

Clinical data were acquired from TCGA and was analyzed by R language software. Kruskal–Wallis rank sum or Wilcoxon rank sum test was used to analyze the correlation between scores and clinical stages.

### Tissue Samples and Immunohistochemistry

Seventy-nine tissue microarrays, including nine normal cervical tissues and 70 cervical squamous cell carcinomas tissues, were purchased from Avilabio, Inc. (Shanxi, China). Informed consents were obtained from all patients before sample collections. The detail clinical.characteristics of the 79 patients were displayed in Table 1. 5 μm tissue sections were obtained from several representative areas of each tumor specimens. They were mounted on glass slides for immunostaining. Immunostaining was carried out by incubation with anti-CCR7 rabbit mAb (1:200; Proteintech) for 2 h at room temperature. The specimen was considered positive if the distinct cytoplasmic staining was observed in >50% of cancer cells.

### PPI Network Construction and Cox Regression Analysis

PPI network was produced by the STRING database and reconstructed using Cytoscape of version 3.7.2 (Cline et al., 2007). Nodes with the confidence of interactive relationships >0.95 were further applied to construct a network. Cox regression analysis was conducted by using R package survival. The top 14 genes were presented in the plot.

### TICs Profile

The TIC abundance profile was estimated by the computational CIBERSORT method (Newman et al., 2015). Only 211 tumor samples with *p* < 0.05 were screened out by quality filtering and applied to the following analysis.

### Gene Set Enrichment Analysis

C7.all.v7.1.symbols and Hallmark gene sets were downloaded from the Molecular Signatures Database^2^. All gene sets were analyzed for GSEA by utilizing the software of gsea-4.0 downloaded from Broad Institute. Only FDR *q* < 0.05 and NOM *p* < 0.05 of gene sets were defined as significant.

## Data Availability Statement

The original contributions presented in the study are included in the article/Supplementary Material, further inquiries can be directed to the corresponding authors.

## Author Contributions

W-JT and P-HF collected the data and wrote the manuscript. JW, TY, Q-FQ, and W-TL visualized the data and reviewed the manuscript. D-LL and W-TL designed the study. All the authors approved the final manuscript.

## Conflict of Interest

The authors declare that the research was conducted in the absence of any commercial or financial relationships that could be construed as a potential conflict of interest.

## Footnotes

1 https://sourceforge.net/projects/estimateproject/

2 http://software.broadinstitute.org/gsea/msigdb/index.jsp

## Supplementary Material

Supplementary Figure 1 Relationship between StromalScore and ImmuneScore and clinical features. **(A–C)** Distribution of StromalScore, ESTIMATEScore, and ImmuneScore in grade. The *p* = 0.75, 0.44, and 0.86, respectively. **(D–F)** Distribution of three types of scores in the T classification (*p* > 0.05 for all three scores). **(G–I)** Distribution of scores in N classification (*p* > 0.05 for all three scores).

Supplementary Table 1 Clinicopathological characteristics of CSCC patients from TCGA.

Supplementary Table 2 TICs co-determined by difference test and correlation test.
