# Supplementary material for: CCR7 Has Potential to Be a Prognosis Marker for Cervical Squamous Cell Carcinoma and an Index for Tumor Microenvironment Change
Source: Front Mol Biosci. 2021 Apr 1;8:583028. doi: 10.3389/fmolb.2021.583028 (PMC8047428; doi:10.3389/fmolb.2021.583028)
Supplement: Supplementary Table 1 — Clinicopathological characteristics of CSCC patients from TCGA. [file Table_1.DOCX]

Supplementary Table 1. Clinicopathological characteristics of CSCC patients from TCGA.

| Clinical characteristics |  | | Total  (253) | | % |
| --- | --- | --- | --- | --- | --- |
| Age at diagnosis (y)  Grade  T classification  N classification | young age (<50)  old age (>=50)  Ⅰ-Ⅱ  Ⅲ-Ⅳ  Unknow  T1  T2  T3  T4  Unknow  N0  N1  Unknow | 146  107  121  104  28  110  59  18  9  57  105  51  97 | | 57.7  42.3  47.8  41.1  11.1  0.43  0.23  0.07  0.04  0.23  0.42  0.20  0.38 | |
