# Supplementary material for: CCR7 Has Potential to Be a Prognosis Marker for Cervical Squamous Cell Carcinoma and an Index for Tumor Microenvironment Change
Source: Front Mol Biosci. 2021 Apr 1;8:583028. doi: 10.3389/fmolb.2021.583028 (PMC8047428; doi:10.3389/fmolb.2021.583028)
Supplement: Supplementary Table 2 — TICs co-determined by difference test and correlation test. [file Table_2.DOCX]

Supplementary Table 2. TICs co-determined by difference test and correlation test

| TICs | Correlation test（p-value） | | Difference test（p-value） |
| --- | --- | --- | --- |
| B cells naive  Plasma cells  T cells CD8  T cells regulatory (Tregs)  NK cells activated  Macrophages M0  Mast cells resting  Mast cells activated | 0.22(0.004)  0.24(0.002)  0.27(<0.001)  0.36(<0.001)  -0.25(0.001)  -0.2(0.008)  0.26(0.001)  -0.34(<0.001) | 0.015  0.005  0.007  <0.001  0.002  0.014  0.014  <0.001 | |
